# Transporting monovalent rotavirus vaccine efficacy estimates to an external target population: a secondary analysis of data from a randomised controlled trial in Malawi

**DOI:** 10.1017/S0950268823000286

**Published:** 2023-02-27

**Authors:** Denise T. St Jean, Jessie K. Edwards, Elizabeth T. Rogawski McQuade, Peyton Thompson, James C. Thomas, Sylvia Becker-Dreps

**Affiliations:** 1 Department of Epidemiology, Gillings School of Global Public Health, University of North Carolina at Chapel Hill, Chapel Hill, North Carolina, USA; 2 Department of Epidemiology, Rollins School of Public Health, Emory University, Atlanta, Georgia, USA; 3 Division of Infectious Diseases, Department of Pediatrics, School of Medicine, University of North Carolina at Chapel Hill, Chapel Hill, North Carolina, USA; 4 Department of Family Medicine, School of Medicine, University of North Carolina at Chapel Hill, Chapel Hill, North Carolina, USA

**Keywords:** Clinical trials, external validity, gastroenteritis, rotavirus, vaccines

## Abstract

Oral rotavirus vaccine efficacy estimates from randomised controlled trials are highly variable across settings. Although the randomised study design increases the likelihood of internal validity of findings, results from trials may not always apply outside the context of the study due to differences between trial participants and the target population. Here, we used a weight-based method to transport results from a monovalent rotavirus vaccine clinical trial conducted in Malawi between 2005 and 2008 to a target population of all trial-eligible children in Malawi, represented by data from the 2015–2016 Malawi Demographic and Health Survey (DHS). We reweighted trial participants to reflect the population characteristics described by the Malawi DHS. Vaccine efficacy was estimated for 1008 trial participants after applying these weights such that they represented trial-eligible children in Malawi. We also conducted subgroup analyses to examine the heterogeneous treatment effects by stunting and tuberculosis vaccination status at enrolment. In the original trial, the estimates of one-year vaccine efficacy against severe rotavirus gastroenteritis and any-severity rotavirus gastroenteritis in Malawi were 49.2% (95% CI 15.6%–70.3%) and 32.1% (95% CI 2.5%–53.1%), respectively. After weighting trial participants to represent all trial-eligible children in Malawi, vaccine efficacy increased to 62.2% (95% CI 35.5%–79.0%) against severe rotavirus gastroenteritis and 38.9% (95% CI 11.4%–58.5%) against any-severity rotavirus gastroenteritis. Rotavirus vaccine efficacy may differ between trial participants and target populations when these two populations differ. Differences in tuberculosis vaccination status between the trial sample and DHS population contributed to varying trial and target population vaccine efficacy estimates.

## Introduction

Rotavirus vaccines are widely used for the prevention of rotavirus gastroenteritis, a major cause of morbidity and mortality among infants and young children worldwide [1]. Phase III, multicentre randomised controlled trials of monovalent rotavirus vaccine (RV1) in Africa [2] and Europe [3] showed that RV1 effectively reduced the risk of rotavirus gastroenteritis in children, but with varying efficacy across locations. As well-designed and properly conducted clinical trials, the RV1 trials have strong internal validity or lack of bias within the study sample [4]. However, findings from the trials may suffer from a lack of external validity or applicability to populations outside of the study who also meet the inclusion criteria.

External validity of RV1 and other oral rotavirus vaccine trials is important given the variable performance exhibited by the vaccine. These variations warrant closer examination of characteristics plausibly associated with both contracting rotavirus and selection into rotavirus trials. Notable characteristics that might threaten external validity for RV1 trials include sociodemographic factors [5], maternal immunity [6, 7], prior medication use [8, 9], breastfeeding and nutritional status [10] and health of the gut microbiome [11]. To date, most studies assessing how the representativeness of the trial sample may affect real-world findings have focused on data collected in high-income settings [12, 13].

The objective of this study was to estimate the real-world effectiveness of RV1 in Malawi by transporting the vaccine efficacy in the GlaxoSmithKline (GSK) RV1 trial in Malawi. Transportability, unlike generalisability, estimates the effect of the treatment in a target population distinct from the study sample [14]. We calculated the expected RV1 vaccine efficacy against severe and any-severity rotavirus gastroenteritis in the present-day Malawian population that would have been eligible for the trial, as represented in the 2015–2016 Malawi Demographic and Health Survey (DHS) [15]. We hypothesised that the estimated vaccine efficacies would differ between the trial sample and the target population due to differences in treatment effect within subgroups of individuals enrolled in the trial.

## Methods

### Study population

We transported results from a Phase III, placebo-controlled RV1 trial conducted in South Africa and Malawi between 2005 and 2008 [Clinical Trial Number: NCT00241644]. The trial has been described in detail in previously published articles [2, 16, 17]. To summarise, healthy infants between 5 and 10 weeks of age residing in the study area were enrolled and randomly assigned to receive three doses of placebo, a placebo dose followed by two vaccine doses or three vaccine doses at approximately 6, 10 and 14 weeks of age. Infants randomised to receive three doses of RV1 were excluded from the analysis.

In the trial, demographic information was collected at baseline. Beginning at study enrolment, study staff made weekly visits to the infant's household to check for gastroenteritis episodes. Caregivers were encouraged to bring the child to local health clinics in the event of illness. Stool samples were collected as soon as possible after symptoms began, but no later than 7 days after onset of gastroenteritis. Samples were analysed for rotavirus antigens using enzyme-linked immunosorbent assays (ELISA) (Meridian Bioscience, Cincinnati, OH, USA) and reverse transcription polymerase chain reaction, followed by reverse hybridisation assay or sequencing in order to differentiate between vaccine and non-vaccine strain genotypes [18]. Follow-up continued until one year of age, with a subset of infants receiving two years of follow-up.

Acute gastroenteritis was defined as the presence of diarrhoea (i.e., three or more stools that were looser than normal within a 24-h period) with or without vomiting. Gastroenteritis episodes were classified as two separate episodes if there was an interval of five or more symptom-free days between the episodes. Rotavirus gastroenteritis was defined as an episode of gastroenteritis occurring at least two weeks after administration of the third dose of study vaccine and a positive ELISA. Severe rotavirus gastroenteritis was defined as an episode of rotavirus gastroenteritis with a score of 11 or greater on the Vesikari scale [19].

We selected six variables that potentially modified the effect of RV1 and may have been associated with selection into the trial based on a review of the substantive literature and data availability: age, sex, receipt of birth dose oral poliovirus vaccine (OPV0) and Bacille Calmette-Guérin (BCG; tuberculosis vaccine), low weight-for-age (i.e., underweight) and low length-for-age (i.e., stunting) [20–24].

### 2015–2016 Malawi DHS data

The target population of interest included children in Malawi who would have been eligible to participate in the GSK trial. To estimate the characteristics of this population, we obtained 2015–2016 Malawi DHS data from the Integrated Public Use Microdata Series DHS portal [25]. The 2015–2016 Malawi DHS was implemented by the National Statistical Office and the Community Health Sciences Unit between October 2015 and February 2016, with a nationally representative sample of over 27 000 households [15]. Based on the trial inclusion criteria, we restricted to children ages 4 to 12 weeks at the time that the DHS was implemented. Anthropometric data were collected for a random sample of one-third of the households. Weight was measured with an electronic flat scale while recumbent length was measured with a ShorrBoard (Olney, MD, USA) measuring board [15]. Each respondent in the DHS was assigned an individual sampling weight to account for difference in sample allocation between districts in Malawi, differential survey response rates and to ensure that survey findings were nationally representative. Sampling weight calculations are described in the Malawi DHS 2015–16 report [15]. Among all eligible DHS children, we selected only those who had complete data for all effect modifiers to be included in our main analysis.

### Statistical analysis

Within the GSK trial, we estimated the one-year vaccine efficacy against rotavirus gastroenteritis in the target population. Vaccine efficacy against both severe and any-severity rotavirus gastroenteritis was calculated using the following formula: vaccine efficacy = (1 – risk ratio (RR)) × 100, where the RR is the cumulative incidence of rotavirus gastroenteritis in the vaccinated group divided by the cumulative incidence of rotavirus gastroenteritis in the placebo group [26]. We used log binomial regression to estimate the RR and 95% confidence intervals (CIs) for vaccine efficacy.

We then estimated vaccine efficacy in the target population by reweighting the trial participants to have the same distribution of effect modifiers as the target population using inverse odds of sampling weights as previously described by Westreich *et al*. [14]. We combined individual data from the GSK trial and the Malawi DHS to estimate the probability of being in the trial, using a weighted multivariable logistic regression model. Independent variables in the model included all effect modifiers of interest and 2-way interaction terms with age and sex. To accurately incorporate the DHS survey weights, trial participants were assigned a weight of 1.0 while survey participants were assigned their DHS sampling weights, as described in Ackerman *et al*. [27].

Next, we computed the inverse odds of inclusion in the trial using the predicted probabilities from the aforementioned model. We assigned individuals who did not participate in the trial a weight of 0. Otherwise, the weight was calculated as the inverse odds of the sampling probability (i.e. the inverse of the ratio of an individual's probability of being in the study sample conditional on *Z*_i_, divided by their probability of not being in the study sample conditional on *Z*_i_) scaled by the marginal odds of being in the trial using the following formula*:* (*P* (*S*_*i*_ = 0 | *Z*_*i*_) /*P* (*S*_*i*_ = 1 |*Z*_*i*_)) × (*P*(*S*_*i*_ = 1)/ *P*(*S*_*i*_ = 0)), where *S*_i_ indicates trial participation and *Z*_i_ indicates a vector of effect modifiers [14].

To explain differences in vaccine efficacy between the target and trial populations, we examined differences in treatment effect within subgroups of trial participants. We limited subgroup analyses to effect modifiers that differed substantially between the GSK sample and the DHS population, defined by an absolute standardised mean difference (ASMD) greater than or equal to 0.20. To determine whether variables were effect measure modifiers on the relative scale, we compared a fully adjusted model including an interaction term between the potential effect modifier and vaccination status to a reduced model with no interaction term. We performed *χ*^2^ tests and conducted a likelihood ratio test (LRT) evaluated at a significance level of 0.10 for each variable. All statistical analyses were performed in R statistical software, version 4.1.0 [28].

### Ethical considerations

This study was approved by the University of North Carolina at Chapel Hill Institutional Review Board (IRB #: 20-2672), GSK Independent Review Panel and the DHS Program.

## Results

### Comparison of characteristics of trial sample, DHS population and target population

The GSK trial included a total of 1773 enrolled children in Malawi and 3168 children enrolled in South Africa; we limited our analysis to the Malawian enrolees. Based on the per-protocol analysis, we included 483 children from the placebo arm and 525 children from the two-dose interventional arm for a total analytic sample of 1008. All children (*n* = 592) from the three-dose arm, 108 children (18%) from the placebo arm and 65 children (11%) from the two-dose arm were excluded from the analysis. Exclusions due to protocol deviations in the placebo and two-dose arm are described in detail in a previously published manuscript [17].

We identified a total of 870 out of 17 386 children in the weighted DHS who would have been eligible for the GSK trial based on age at the time of the survey. We excluded an additional 642 who did not have complete anthropometric data, for a complete case subset of 228. The final analytical sample was representative of the full DHS sample (Supplemental Table S1). Anthropometric data were collected from only a subset of children in the DHS, and therefore were missing by design.

Table 1 presents the distribution of effect modifiers in the DHS population and GSK trial, both before and after reweighting the trial participants to resemble the DHS population. While some variables, such as underweight status and receipt of OPV0, were distributed similarly between the DHS population and the GSK trial sample, other variables differed between the two populations. Reweighting the GSK trial data to represent the target population resulted in better balance among effect modifiers and reduced ASMD among all variables. Henceforth, the GSK trial data after reweighting will be referred to as the ‘target population’.

**Table 1.** Distribution of effect modifiers before and after weighting by inverse odds of sampling weights in the GlaxoSmithKline (GSK) RV1 trial in Malawi, 2005–2008 and the 2015–2016 Malawi Demographic and Health Survey (DHS)

Effect modifier	DHS (*n* = 228)		GSK Trial (*n* = 1008)			GSK trial data weighted by inverse odds of sampling		
	*n* (%)	Mean [s.d.]	*n* (%)	Mean [s.d.]	ASMD	*n* (%)	Mean [s.d.]	ASMD
Age, months		1.13 [0.75]		1.04 [0.19]	0.17		1.05 [0.22]	0.14
Female sex	121 (52.9)		475 (47.1)		0.12	469 (52.3)		0.01
Underweight^a^	10 (4.5)		42 (4.2)		0.02	37 (4.1)		0.02
Stunted^b^	43 (18.8)		397 (39.4)		0.46	170 (19.0)		<0.005
Received OPV0	171 (75.1)		717 (71.1)		0.09	653 (72.9)		0.05
Received BCG	204 (89.4)		824 (81.7)		0.22	793 (88.6)		0.03

Abbreviations: ASMD, absolute standardised mean differences; BCG, bacille Calmette-Guérin; OPV0, birth dose of oral poliovirus vaccine; RV1, monovalent rotavirus vaccine; s.d., standard deviation.

a Average weight-for-age *Z* score < − 2 s.d. from the WHO Child Growth Standards median.

b Average length-for-age *Z* score < − 2 s.d. from the WHO Child Growth Standards median.

### Comparison of treatment effects in the trial and target population

**Fig. 1.**
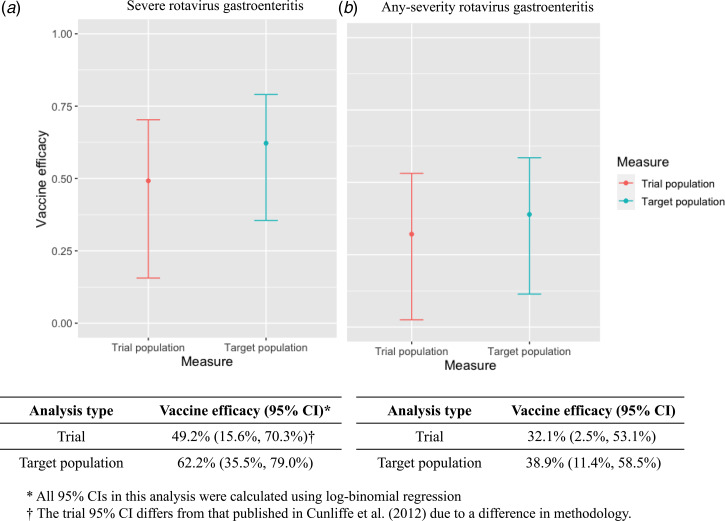
Comparison of trial and target population vaccine efficacy and 95% CIs for (a) severe rotavirus gastroenteritis and (b) any-severity gastroenteritis.

Table 2 presents the RRs with 95% CIs for the effect of RV1 on rotavirus gastroenteritis in the trial and target populations. In the trial, RV1 was associated with a lower risk of severe and any-severity rotavirus gastroenteritis compared to placebo. The original per-protocol analysis of the GSK trial found a one-year vaccine efficacy of 49.2% (95% CI 15.6–70.3) against severe rotavirus gastroenteritis in Malawi [17] and 32.1% (95% CI 2.5%–53.1%) against any-severity rotavirus gastroenteritis (Fig. 1). After weighting to represent the target population, vaccine efficacy increased to 62.2% (95% CI 35.5%–79.0%) for severe rotavirus gastroenteritis and 38.9% (95% CI 11.4%–58.5%) for any-severity rotavirus gastroenteritis. Compared with the treatment effect observed in the trial, the expected vaccine efficacy in the target population was 13 percentage points higher for severe rotavirus gastroenteritis. The vaccine efficacies and precision of the estimates for any-severity rotavirus gastroenteritis were similar in the trial and target populations.

**Table 2.** One-year risk ratios and vaccine efficacy of severe and any-severity rotavirus gastroenteritis (RVGE) in the GlaxoSmithKline (GSK) RV1 trial in Malawi, 2005–2008 and the target population

Analysis type	Number of children	Risk of RVGE	Risk ratio (95% CI)	Vaccine efficacy (95% CI)
*Severe RVGE*				
GSK Trial				
Placebo	483	0.08	(ref)	1.0 (ref)
RV1	525	0.04	0.51 (0.30–0.84)	0.49 (0.16–0.70)
Target population^a^				
Placebo	439	0.10	1.0 (ref)	1.0 (ref)
RV1	456	0.04	0.38 (0.21–0.64)	0.62 (0.36–0.79)
*Any RVGE*				
GSK Trial				
Placebo	483	0.13	1.0 (ref)	1.0 (ref)
RV1	525	0.09	0.68 (0.47–0.97)	0.32 (0.03–0.53)
Target population^a^				
Placebo	439	0.14	1.0 (ref)	1.0 (ref)
RV1	456	0.09	0.61 (0.42–0.89)	0.39 (0.11–0.59)

Abbreviations: RV1, monovalent rotavirus vaccine; RVGE, rotavirus gastroenteritis.

a The target population is represented by the GSK trial data weighted by inverse odds of sampling.

### Subgroup analysis in the target population

We identified two variables whose distributions differed between the trial and DHS (ASMD ≥ 0.20) to assess for treatment effect heterogeneity: receipt of BCG and stunting. We found that for severe rotavirus gastroenteritis, children who had received BCG demonstrated larger treatment effects as compared with those who had not received BCG on the relative risk scale (RR = 0.42 *vs.* 1.08, *P* = 0.10; Table 3). No such differences by BCG receipt were observed for any-severity rotavirus gastroenteritis (RR = 0.64 *vs.* 0.82, *P* = 0.34). There were also no large differences in the benefit from RV1 for severe rotavirus (RR = 0.46 *vs.* 0.62, *P* = 0.78) or any-severity rotavirus gastroenteritis (RR = 0.62 *vs.* 0.81, *P* = 0.43) in those who were stunted *vs.* not stunted.

**Table 3.** Subgroup analysis by stunted status and BCG vaccination status^a^, using stratum-specific one-year risk ratios (RR) for (A) severe and (B) any-severity rotavirus gastroenteritis (RVGE) and likelihood ratio tests

A. Severe RVGE		RV1	Placebo		*LRT of homogeneity*	
Modification variable	Stratum	Severe RVGE/Total RVGE	Severe RVGE/Total RVGE	RR (95% CI) for RVGE within strata of potential modifier	*χ*^2^	*P* value
Stunting^b^	No	14/365	37/361	0.46 (0.24–0.88)	0.08	0.78
	Yes	2/91	5/78	0.62 (0.26–1.51)		
Completed BCG	No	3/51	3/51	1.08 (0.34–3.40)	2.73	**0.098**
	Yes	13/405	38/387	0.42 (0.23–0.76)		

B. Any-severity RVGE		RV1	Placebo		*LRT of homogeneity*	
Modification variable	Stratum	Any RVGE/Total RVGE	Any RVGE/Total RVGE	RR (95% CI) for RVGE within strata of potential modifier	*χ*^2^	*P* value
Stunting^b^	No	32/365	54/361	0.62 (0.39–0.98)	0.61	0.43
	Yes	8/92	8/78	0.81 (0.44–1.50)		
Completed BCG	No	7/51	8/52	0.82 (0.38–1.77)	0.90	0.34
	Yes	32/405	54/388	0.64 (0.42–0.97)		

Abbreviations: BCG, bacille Calmette-Guérin; CI, confidence interval; LRT, likelihood ratio test; RR, risk ratio; RVGE, rotavirus gastroenteritis.

a Effect modifiers with an absolute standardised mean difference (ASMD) ≥0.20 were selected for subgroup analyses.

b Average length-for-age *Z* score < − 2 s.d. from the WHO Child Growth Standards median.

## Discussion

In this study, we showed that transporting the GSK RV1 trial results to the Malawi target population who would have been eligible to enrol in the trial led to higher estimated vaccine efficacy for RV1 against severe and any-severity rotavirus gastroenteritis. We found that the effect of RV1 on reducing severe and any-severity rotavirus gastroenteritis increased after reweighting the trial population, possibly due to effect measure modification by receipt of BCG. Reweighting the trial population to the Malawi DHS allowed us to estimate the treatment effect in the target population while still benefitting from the advantages of randomisation. Recent studies by Stuart *et al*. [29] and Susukida *et al*. [13] apply a similar weight-based approach to generalise results of a behavioural intervention in schools and community substance use disorder treatment, respectively. To our knowledge, this study is the first to use this approach to estimate target population effects to a vaccine study using data from a low-income country.

Our findings are consistent with a previous observational study of the impact of RV1 introduction in Malawi published in 2015, which estimated 64% vaccine efficacy of RV1 against severe rotavirus gastroenteritis [30]. The higher vaccine efficacy estimated in the target population can be explained, in part, by a greater number of children in the target population receiving the BCG vaccine. The Malawi Expanded Program on Immunization recommends that BCG be administered at birth or first contact. Timely administration of birth vaccines is especially challenging in sub-Saharan Africa due to limited awareness of birth vaccines among healthcare workers and mothers, poor communication and coordination between healthcare workers and mothers and challenges of reaching infants born outside of health care facilities [31, 32]. Greater benefits of RV1 against severe and any-severity rotavirus gastroenteritis were observed in the GSK trial for children who were vaccinated against BCG compared to those who were not. Receipt of the BCG vaccine could induce anti-viral effects [33] or could be indicative of other sociodemographic indicators, such as socioeconomic status and maternal education, that influence health-seeking behaviours and reduce rotavirus risk [34, 35]. Though concomitant administration of OPV with RV1 has been associated with impaired seroconversion [24, 36], OPV0 receipt was similar in both the trial and target populations and therefore not assessed as an effect modifier in this study.

Although anthropometric measures commonly associated with altered growth, such as stunting, wasting and being underweight, are considered risk factors for acute gastroenteritis, evidence surrounding the relationship between malnutrition and rotavirus gastroenteritis is conflicting [20, 21]. While we did not have sufficient evidence to suggest modification of the relationship between rotavirus gastroenteritis by stunting status in our study, children who were stunted experienced a higher risk of rotavirus gastroenteritis. Effect measure modification by anthropometric variables can vary across settings. Two longitudinal studies in Bangladesh observed a significant positive association between appropriate weight gain and rotavirus infections in children under five [20, 21]. On the other hand, a secondary analysis of RV1 efficacy against rotavirus gastroenteritis in Brazil, Mexico and Venezuela determined that vaccine efficacies were similar between well-nourished and malnourished children [37]. Nutrition status is often closely related to diet and breastfeeding status, which also provides strong protection against rotavirus infections. Future studies are needed to further explore the relationship between diet and breastfeeding status, nutrition status and vaccine response in varied settings.

While we used the most recent Malawi DHS in our analysis, the ideal dataset to represent the target population can vary based on data availability and the research objective. For those who aim to inform decision-making going forward, the best dataset is the one that reflects the most current information on the target population. However, to simply assess the generalisability of findings to all trial-eligible participants at the time of the trial, the best dataset is one from a similar time period as the clinical trial.

The study was limited by the low number of characteristics measured in both the trial sample and the DHS population. Specifically, few participant characteristics were collected in the trial sample. Therefore, it is likely that sample weights calculated in this study did not take into account other important characteristics, such as socioeconomic status, breastfeeding status, prior medication use and urban *vs.* rural residence, that may differ between the trial sample and target population and may thereby modify the effect of RV1. Also, due to the small sample size after applying the trial exclusion criteria and additionally excluding individuals with missing data on effect modifiers, the DHS population may not have adequately reflected the true target population. However, because most of the data were missing completely at random [38] based on anthropometric data collection, it is unlikely that the complete case analysis introduced bias into the results. Another limitation of this analysis is that it does not account for indirect effects of rotavirus vaccines [39], which we know provide an additional benefit. And finally, the primary goal of the original GSK trial was not to assess treatment effect heterogeneity. Consequently, the subgroup analyses conducted for this study were not adequately powered to detect differences across subgroups of children.

Despite these limitations, results from this study provide insight into whether deviations in trial sample representativeness from target populations influence treatment estimates for rotavirus vaccine trials. It is critical for future vaccine studies to place greater emphasis on external validity, particularly because a primary goal of clinical trials is to provide evidence about vaccinations that can be disseminated globally. This issue was recently brought to the forefront during recruitment for large-scale trials by Pfizer, Moderna and Johnson & Johnson in the development of vaccines against SARS-CoV-2 [40]. Representative individual-level data from a target population is not always easy to come across, especially outside of high-resource settings in North America and Europe. In the absence of individual-level data, methods that utilise aggregate data such as simulations, weighting using the method of moments [41], poststratification [29] and expected absolute risk reduction [42] can be implemented. In a comparison of methods to generalise clinical trials to real-world settings, Hong *et al*. found that methods using aggregate data for the target population were comparable with the gold-standard approach used in this analysis [12].

In conclusion, our study demonstrates the possibility of transporting vaccine trial results to a target population using individual-level data from a complex survey to represent the target population. Given that clinical trials are usually costly, time-consuming and limited to a small number of study sites, it can be helpful to understand the applicability of the findings to different populations and settings. Recruiting diverse trial participants, collecting detailed data on potential effect measure modifiers and employing quantitative methods such as those described in this paper may provide policy makers with more realistic estimates of the benefits of vaccines to support public health planning during vaccine rollouts.

## Data Availability

The data underlying this analysis are available by request through a third party, ClinicalStudyDatarequest.com. The data request must include a research proposal. An Independent Review Panel will review all research proposals and provide notification if access has been granted or additional project information is needed before access can be granted.

## Supplementary material

For supplementary material accompanying this paper visit http://doi.org/10.1017/S0950268823000286. click here to view supplementary material
