# Supplementary material for: Transporting monovalent rotavirus vaccine efficacy estimates to an external target population: a secondary analysis of data from a randomised controlled trial in Malawi
Source: Epidemiol Infect. 2023 Feb 27;151:e49. doi: 10.1017/S0950268823000286 (PMC10052556; doi:10.1017/S0950268823000286)
Supplement: Supplementary file 1 [file S0950268823000286sup001.docx]

**Supplemental Table 1.** Baseline characteristics among the full sample (n=870) and complete case subset (n=228) of 2015-2016 Malawi Demographic and Health Survey (DHS) participants

| **Effect modifier** | **Full Sample DHS**  **(n=870)** | | **Complete Case DHS**  **(n=228)** | |
| --- | --- | --- | --- | --- |
|  | **n (%)** | **mean [SD]** | **n (%)** | **mean [SD]** |
| Age, months |  | 1.04 [0.81] |  | 1.13 [0.75] |
| Female sex | 455 (52.3) |  | 121 (52.9) |  |
| Underweight^a^ | 10 (4.4) |  | 10 (4.5) |  |
| Stunted^b^ | 43 (18.7) |  | 43 (18.8) |  |
| Received OPV0 | 634 (75.5) |  | 171 (75.1) |  |
| Received BCG | 726 (86.6) |  | 204 (89.4) |  |

Abbreviations: BCG, bacille Calmette-Guérin; OPV0, birth dose of oral poliovirus vaccine; SD, standard deviation;

^a^ Average weight-for-age Z score < −2 SD from the WHO Child Growth Standards median

^b^ Average length-for-age Z score < −2 SD from the WHO Child Growth Standards median
